# A comparative study of parasites in three latrines from Medieval and Renaissance Brussels, Belgium (14th–17th centuries)

**DOI:** 10.1017/S0031182020001298

**Published:** 2020-11

**Authors:** Anna Graff, Emma Bennion-Pedley, Ariadin K. Jones, Marissa L. Ledger, Koen Deforce, Ann Degraeve, Sylvie Byl, Piers D. Mitchell

**Affiliations:** 1 Department of Archaeology, University of Cambridge, The Henry Wellcome Building, Fitzwilliam Street, Cambridge CB2 1QH, UK; 2 Royal Belgian Institute of Natural Sciences, Vautierstraat 29, 1000 Brussels, Belgium; 3 Brussel Stedenbouw en Erfgoed, Kunstberg 10-13, 1000 Brussels, Belgium; 4 Centre de Recherches en Archéologie et Patrimoine, Université libre de Bruxelles, Avenue F.D. Roosevelt 50, B-1050 Brussels, Belgium

**Keywords:** *Ascaris*, *Capillaria*, *Dicrocoelium*, *Entamoeba histolytica*, *Fasciola*, *Giardia duodenalis*, Palaeoparasitology, *Taenia*, *Trichuris*

## Abstract

The aim of this study is to determine the species of parasite that infected the population of Brussels during the Medieval and Renaissance periods, and determine if there was notable variation between different households within the city. We compared multiple sediment layers from cesspits beneath three different latrines dating from the 14th–17th centuries. Helminths and protozoa were detected using microscopy and enzyme-linked immunosorbent assay (ELISA). We identified *Ascaris* sp., *Capillaria* sp., *Dicrocoelium dendriticum*, *Entamoeba histolytica*, *Fasciola hepatica*, *Giardia duodenalis*, *Taenia* sp. and *Trichuris* sp. in Medieval samples, and continuing presence of *Ascaris* sp., *D. dendriticum*, *F. hepatica*, *G. duodenalis* and *Trichuris* sp. into the Renaissance. While some variation existed between households, there was a broadly consistent pattern with the domination of species spread by fecal contamination of food and drink (whipworm, roundworm and protozoa that cause dysentery). These data allow us to explore diet and hygiene, together with routes for the spread of fecal–oral parasites. Key factors explaining our findings are manuring practices with human excrement in market gardens, and flooding of the polluted River Senne during the 14th–17th centuries.

## Introduction

In the late Medieval and Renaissance periods, the Low Countries (now Belgium and the Netherlands) were a key European power for maritime trade, banking and business. Cities such as Bruges, Ghent and Brussels facilitated the movement of goods between Britain, Scandinavia and the Baltic regions in the north to the Mediterranean cultures and Arab world in the south. With trade and migration comes the potential for infectious disease spread, and as population centres increase in size and density, so does the ease of spread of parasites.

Brussels at this time had established itself as a political and economic centre of the region (Charruadas and Dessouroux, 2005). It counted 20 000 inhabitants in 1300 and 26 000 in 1400, with population density increasing in the decades to come (Charruadas *et al*., 2017). From the 14th century onward, substantial urbanization took place, with considerable artisan activities and the emergence of brick and stone buildings (Vannieuwenhuyze *et al*., 2015). In recent decades, there have been a number of excavations of cesspits in northern Belgium (De Clercq *et al*., 2007; Troubleyn *et al*., 2009). The main focus of this work so far has been on dietary reconstruction based on the analysis of macro and micro botanical remains (such as seeds and pollen) and animal bones found in the cesspits, site structure and pottery analyses. Diversification of plant and animal remains found in archaeological sediments from the late Medieval period compared to earlier time periods suggest increasing trade relations with distant regions with warmer climates (Charruadas *et al*., 2017). From the late Renaissance to the Early Modern Period (16th–17th century CE), the Low Countries entered into an era marked by robust trends towards political, economic and urban development, as embodied by the city of Brussels (Fernandes *et al*., 2005; Rocha *et al*., 2006; Speleers and van der Valk, 2017).

The aim of this study is to determine the species of parasite that infected the population of Brussels during the late Medieval and Renaissance periods, in order to help us understand the health consequences of this period of mercantile expansion. While some promising parasite research has been undertaken at archaeological sites in Belgium before, these have typically focussed on individual locations. Some analyse sediment from cesspits that contain the comingled feces of different people, while others study coprolites, which are the preserved remains of a defecation event from a single person (Gonçalves *et al*., 2004; Rocha *et al*., 2006; Rocha and Serra-Freire, 2009; Appelt *et al*., 2014), or the intestinal content residues associated with burials (Deforce *et al*., 2015; Rácz *et al*., 2015). While a number focussed their attention on the parasite remains, others primarily focussed on pollen and other plant remains, noting the parasites eggs as an incidental finding (Troubleyn *et al*., 2009; Deforce, 2010). It is not always easy to fully compare the results found if they use different source material, varying methods of analysis and present their data in different ways. If we are able to study a number of different latrines from the same city, using the same techniques of analysis, we can interrogate the data in a more nuanced way. Hence, we have employed a comparative analysis that allows us to assess for the first time how much variation there may have been in parasite species and egg densities in different households in this part of the Low Countries, and also look for any change in infection between the Medieval period and the Renaissance.

## Materials and methods

### Materials

**Fig. 1.**
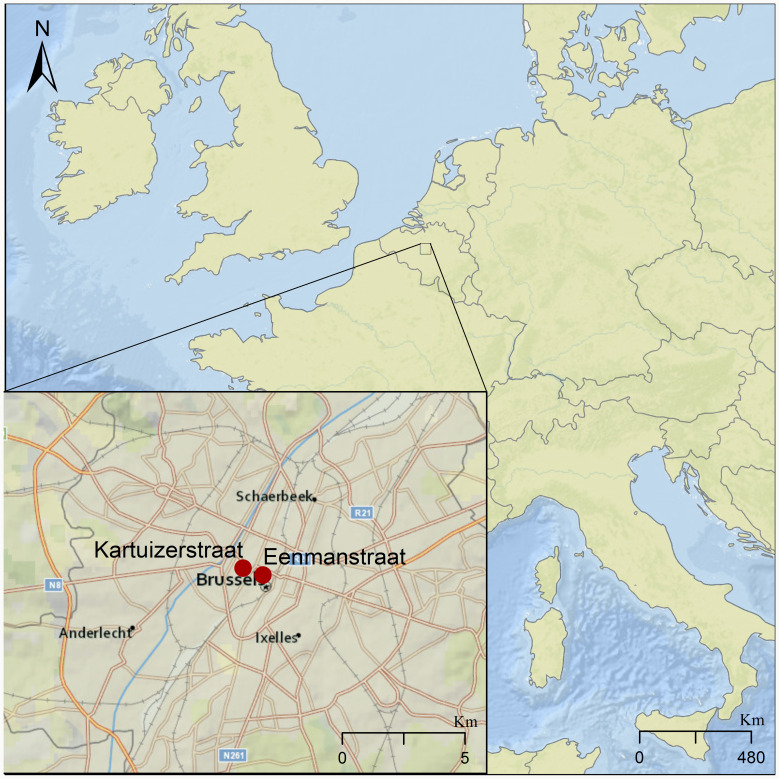
Map of Brussels showing the two excavation sites in red where the analysed latrines were found, marked by red dots in the inset. The larger site to the east is where excavation BR166-03 was conducted, in which Cesspit 1 was excavated. The site to the west represents excavation site BR-111, giving rise to Cesspits 2 and 3.

We tested for the presence of intestinal parasites in eight sediment samples dated between the 14th and 17th centuries. These samples came from three different latrines identified at excavation sites located about 500 m apart in the city centre of Brussels (Fig. 1). These sites are in Eénmanstraat (BR0166-03) and Kartuizerstraat (BR111). Samples from four layers of one brick-lined cesspit (14th–15th c.) were collected from Eénmanstraat. From Kartuizerstraat samples from two different cesspits were collected. One cesspit was unlined (14th–15th c.) and two were brick-lined (14th–15th and 15th–17th c.).

The first latrine considered was a brick-lined cesspit from Eénmanstraat/rue d'Une Personne (latrine US 4067; henceforth: Cesspit 1) showing clearly identifiable stratigraphic layers *(unités stratigraphiques*, US). It was excavated in 2014 by the CReA-Patrimoine (Université libre de Bruxelles) and the Société royale d'Archéologie de Bruxelles in Brussels. The site is known to have represented a bustling artisan and commercial centre in the 14th–15th centuries with opulent butcheries, breweries and bakeries (Billen, 1997). Excavation reports detail that Cesspit 1 was abandoned and partially demolished to enable the construction of a cellar in the 16th century, so must predate this. Two samples were collected and studied from each of two different layers; the lower layer US4139 and subsequent, marginally younger layer US4138. Both layers were composed of organic material and dated to the 14th–15th c. CE based on pottery and sandstone material recovered from the later layer.

The second latrine studied was a 14th–15th century unlined cesspit from Kartuizerstraat (henceforth: Cesspit 2), which also showed defined stratigraphic layers. It was excavated in 2010 by the archaeological team of the Brussels-Capital Region (Departement Archeologisch Erfgoed, Gewestelijke Overheidsdienst Brussel) from the foundations of the art nouveau building, the Café Greenwich, in Brussels Kartuizerstraat (excavation code BR111). There were six layers in total, containing very few archaeological artefacts, other than a few ceramic sherds dating from between the end of the 14th c. and the beginning of the 15th c. AD. Radiocarbon dating (calibrated with Oxcal v 4.3.2. Bronk Ramsey 2017) also produced dates spanning this time period. Parasitological analysis was carried out on three separate layers: US20-134, US23-45 and US24-122.

The third latrine considered was another brick-lined cesspit (henceforth: Cesspit 3), which was also excavated as a part of the project in the Kartuizerstraat (BR111). Similar to the other cesspit from this site, the initial excavation yielded few artefacts within the fill of the cesspit, with the exception of three stoneware vessels from the US39 layer. Parasitological analysis was carried out on one layer from this cesspit US 33-15, dating between the middle of the 15th c. – the first half of the 17th c. Both of the cesspits from BR111 were considerably waterlogged, which preserved the organic macrobotanical remains well enough to be used for radiocarbon dating.

### Microscopy

The samples for this study were processed at the Ancient Parasites Laboratory at the University of Cambridge following our published procedures and methodologies (Anastasiou and Mitchell, 2013*a*). From each of the eight samples, a 0.2 g subsample was disaggregated in 0.5% trisodium phosphate to form an aqueous suspension for a minimum of 2 h. This suspension was then filtered through 300, 160 and 20 *μ*m microsieves stacked with descending mesh size. Thus far, recovered helminth eggs from northern Europe have ranged from 30 to 150 *μ*m. In consequence, the material recovered from the 20 *μ*m sieve would contain all relevant helminth eggs. Once the sample was centrifuged, the remaining liquid was removed and the pellet mixed with glycerol to view with microscopy at 400 ×  magnification. Helminth eggs were identified by size and morphological features outlined in reference texts (Gunn and Pitt, 2012; Ash and Orihel, 2015; Garcia, 2016; Mehlhorn, 2016; World Health Organization, 2019) and previous published paleoparasitological literature. Limitations to the microscopic examination of intestinal parasites include where more than one parasite produces morphologically similar eggs (e.g. beef and pork tapeworm, human and pig whipworm), and where species produce fragile eggs so if damaged or distorted over archaeological time they may not be identifiable (e.g. pinworm and hookworm).

### ELISA

The enzyme-linked immunosorbent assay (ELISA) testing employed commercially available kits produced by TECHLAB^®^. The ELISA kits which were designed to detect antigens from *Cryptosporidum* spp., *E. histolytica* and *G. duodenalis* have been successfully used on archaeological material for many years (Gonçalves *et al*., 2002; Le Bailly and Bouchet, 2006, 2015; Mitchell *et al*., 2008). These test kits have been found to have 98–100% sensitivity and specificity in modern clinical trials of fresh feces (Sharp *et al*., 2001; Verkerke *et al*., 2015). For sample preparation, subsamples of 1 g were taken from each sample. They were once again disaggregated with 0.5% trisodium phosphate and sieved using 300, 160 and 20 *μ*m microsieves. The material used for these tests was collected from the liquid that passed through all the sieves, hence containing material smaller than 20 *μ*m. This is because the cysts and oocysts of these three species of protozoa are all smaller than 20 *μ*m. Each sample was tested in eight wells on the ELISA plates, thereby having an entire column dedicated to each sample. Each plate also had a negative and positive control. Positive controls were provided with the kits. Furthermore, each sample underwent two rounds of testing on separate subsamples on two separate days in order to ensure the replicability of the results. Congruent with previous studies administered in our laboratory, the subsequent absorbance values used to determine if a well is positive or negative were measured using a BioTek Synergy HT Multi-Mode Microplate Reader. Limitations to the use of ELISA in excavated cesspit samples are that over archaeological time many of the fragile cysts originally present may be destroyed by soil fungi and insects, so insufficient numbers may be present in any one ELISA well to trigger a positive result in the test, and also that the antigen proteins detected in the test could change their structure due to the soil environment, again leading to a false-negative test. Therefore, the sensitivity of each test is likely to be lower than in modern clinical studies.

## Results

### Microscopy

**Fig. 2.**
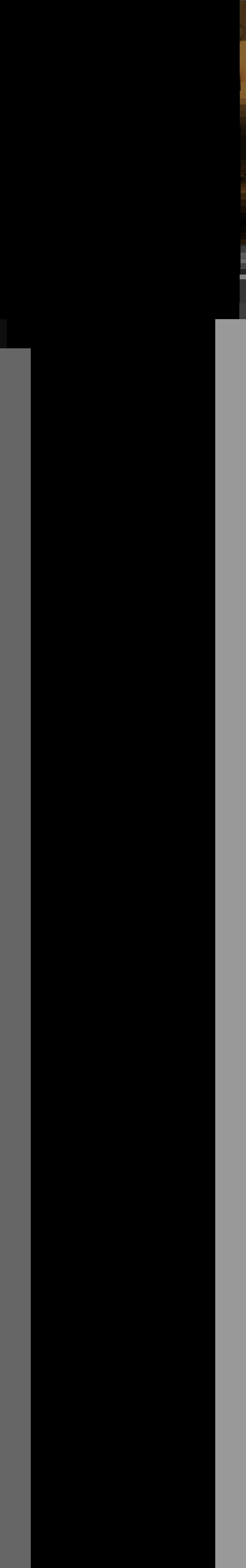
(A) unfertilized *Ascaris* sp. egg from Cesspit 1 (dimensions 81 *μ*m × 41 *μ*m). (B) Fertilized *Ascaris* sp. egg from Cesspit 1 (dimensions 64 *μ*m × 45 *μ*m). (C) *Fasciola hepatica* egg with intact operculum, originating from Cesspit 3 (dimensions 141 *μ*m × 71*μ*m). (D) *Taenia* sp. egg from Cesspit 1 with dimensions 36 *μ*m × 34 *μ*m. (E) *Taenia* sp. egg with visible hooks in the oncosphere (dimensions: 38 *μ*m × 34 *μ*m). (F) *Dicrocoelium dendriticum* egg from Cesspit 3 (dimensions 44 *μ*m × 26 *μ*m) without operculum, from Cesspit 1 (dimensions 36 *μ*m × 23 *μ*m). (G) *Capillaria* sp. egg from Cesspit 2 showing the characteristic punctuated surface coat (dimensions 44 *μ*m × 25 *μ*m). (F) *Capillaria* sp. egg showing the thick wall and elongated shape (dimension 62 *μ*m × 28 *μ*m). (I) *Trichuris trichiura* egg from Cesspit 2 (dimensions 55 *μ*m × 25 *μ*m). Black bars indicate 20 *μ*m.

Across samples and cesspits, large numbers of well-preserved parasite eggs were found. In total, six genera of helminths were identified: *Ascaris*, *Capillaria*, *Dicrocoelium*, *Fasciola*, *Taenia* and *Trichuris* (Table 1, Fig. 2). High concentrations of the fecal-orally transmitted parasites roundworm (*Ascaris* sp.) and whipworm (*Trichuris* sp.) were found in all cesspits (up to 14 555 eggs g^−1^ for *Ascaris* and 39 640 eggs g^−1^ for *Trichuris*). Zoonotic flukes of the genera *Dicrocoelium* and *Fasciola* were additionally recovered from Cesspits 1 and 3 in lower concentrations. Cesspit 1 also contained tapeworm eggs of the genus *Taenia*; and eggs from the nematode *Capillaria* sp. was found in Cesspit 2.

**Table 1.** Summary of helminth eggs and pathogenic protozoa found in each sample analysed from the three cesspits

	Helminth eggs (presence, eggs g^−1^)						Protozoa (presence)		
	*Ascaris*	*Capillaria*	*Dicrocoelium*	*Fasciola*	*Taenia*	*Trichuris*	*Cryptosporidium*	*Entamoeba*	*Giardia*
Cesspit 1 14th–15th c.	X	–	X	X	X	X	–	X	X
US4138(379)	9985	–	40	5	–	7750	–	–	+
US4138(480)	13 420	–	20	–	5	1750	–	+	+
US4139(454)	5475	–	10	–	–	4925	–	+	+
US4139(457)	7020	–	–	5	10	39 640	–	–	–
Cesspit 2 14th–15th c.	X	X	–	–	–	X	–	X	X
US20-134	4230	–	–	–	–	27 790	–	+	+
US23-45	3456	10	–	–	–	13 200	–	–	+
US24-122	20	–	–	–	–	75	ELISA not carried out		
Cesspit 3 15th–17th c.	X	–	X	X	–	X	–	–	X
US33-15	14 555	–	10	10	–	5285	–	–	+

*Notes*: For each cesspit sample, the concentration of eggs is given in eggs g^−1^. Presence of protozoa is recorded as positive (+) or negative (-). The parasite eggs present are indicated with an X as a summary for each sample.

**Fig. 3.**
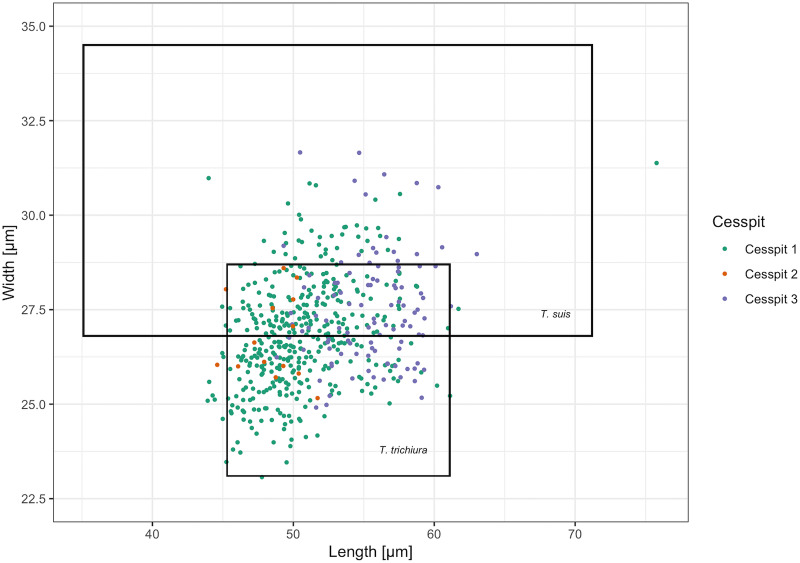
Scatter plot of the measured *Trichuris* spp. egg dimensions across samples including the size ranges for *T. trichiura* and *T. suis* reported by Beer (1976). Eggs with and without preserved polar plugs are included. The cesspit of origin is specified by colour for each egg.

Whipworm eggs (*Trichuris* sp.) were the most common helminth found in Cesspits 1 and 2 and the second most common in Cesspit 3. Human and pig whipworm eggs have overlapping size ranges, with human whipworm being smaller than pig whipworm (Beer, 1976). The mean length of whipworm eggs with preserved polar plugs in each of the three cesspits was 56.4 *μ*m (s.d. 2.5), 53.6 *μ*m (s.d. 2.7) and 56.9 *μ*m (s.d. 2.2), respectively. The mean widths were 27.0 *μ*m (s.d. 1.2), 26.4 *μ*m (s.d. 1.4) and 27.5 *μ*m (s.d. 1.3). Figure 3 shows how these measurements mostly fall within the size ranges for human whipworm (*T. trichiura*) or the pig whipworm (*T. suis*), although some outliers may potentially represent instances of the longer dog or sheep whipworm (*T. vulpis* or *T. ovis*) (Mehlhorn, 2016). Given that all eggs were retrieved from human cesspits, the vast majority of eggs likely represent *T. trichiura.*

Roundworm (*Ascaris* sp.) eggs were the most numerous eggs found in Cesspit 3, and second most common in Cesspits 1 and 2. They were found in both their fertilized and unfertilized forms. Similarly, to *Trichuris* sp., the dimensions of *Ascaris* sp. vary according to the species: 45–75 × 35–50 *μ*m for human roundworm (*A. lumbricoides*) and 50–70 × 40–60 *μ*m for pig roundworm (*A. suum*). There is considerable overlap in these dimensions, making it difficult to reliably distinguish between the two species. As the two species of roundworm are almost morphologically identical and they are so genetically similar that each species can infect both humans and pigs (Betson *et al*., 2014), we did not attempt to identify them to the species level. However, again, due to their presence in human cesspits, it is likely that they represent human infections.

Two taxa of liver flukes were also found in the cesspits. Both of these liver flukes, *Dicrocoelium* sp. and *Fasciola* sp., were identified in Cesspits 1 and 3. While *F. hepatica* and *F. gigantica* appear very similar on microscopy, the eggs recovered are most likely from *F. hepatica*, as it is endemic to Europe and *F. gigantica* is not. For the specific dimensions of eggs recovered from the cesspits, see Table 2.

**Table 2.** Dimensions (mean, standard deviation and range) in *μ*m for the length and width of the species of parasite eggs identified in Cesspit 1, Cesspit 2 and Cesspit 3

			Length			Width		
	Cesspit	*n*	Mean	Std. Dev	Range	Mean	Std. Dev.	Range
*Ascaris* sp., fertilised								
With mamillated coat	1	151	66.6	5.1	53.0–83.0	48.7	3.0	38.4–55.6
	2	166	65.6	4.6	54.3–81.6	49.4	4.1	40.4–62.1
	3	81	67.7	4.6	56.6–78.7	53.6	4.2	45.1–61.6
Without mamillated coat	1	169	64.1	5.2	48.8–76.6	46.1	2.6	35.3–52.9
	2	15	62.1	3.0	58.1–72.0	49.4	2.8	39.0–49.2
	3	2	62.9	0.9	62.2–63.5	46.6	0.1	46.5–46.7
*Ascaris* sp., unfertilised								
With mamillated coat	1	49	86.6	3.7	80.8–95.6	44.6	2.8	40.2–53.6
	2	22	76.4	6.9	62.6–92.8	44.1	6.2	37.8–58.0
	3	28	85.1	5.8	77.1–96.4	48.2	4.5	40.4–59.0
Without mamillated coat	1	31	86.9	4.2	80.2–94.4	42.5	1.9	39.4–49.4
*Capillaria* sp.								
Without polar plugs	2	2	53.4	12.7	44.4–62.1	26.5	1.6	25.4–28.3
*Dicrocoelium* sp.								
With operculum	1	8	38.8	3.7	33.2–43.9	25.4	1.6	22.2–27.3
	2	0	–	–	–	–	–	–
	3	1	43.6	NA	NA	26.3	NA	NA
Without operculum	1	4	37.9	2.4	35.5–41.2	24.2	1.7	22.6–26.5
	2	0	–	–	–	–	–	–
	3	1	41.7	NA	NA	26.1	NA	NA
*Fasciola* sp.								
With operculum	3	1	141.4	NA	NA	71.2	NA	NA
Without operculum	1	1	129.9	NA	NA	84.4	NA	NA
	3	1	130.7	NA	NA	69.8	NA	NA
*Taenia* sp.								
	1	3	36.2	1.4	34.7–37.5	34.1	0.3	33.8–34.4
*Trichuris* sp.								
With 2 polar plugs	1	40	56.4	2.5	51.2–61.7	27.0	1.2	25.0–30.6
	2	27	53.6	2.7	44.6–57.8	26.4	1.4	23.2–28.5
	3	67	56.9	2.2	51.1–63.0	27.5	1.3	25.2–31.1
							
With 1 polar plug	1	51	53.3	1.7	49.4–56.6	27.8	1.3	25.5–30.8
	2	12	51.7	2.0	46.2–55.9	25.9	0.9	24.7–27.6
	3	24	53.9	2.5	49.7–59.3	27.1	1.4	24.9–30.5
Without polar plugs	1	309	49.5	2.9	43.9–75.8	26.6	1.4	23.1–31.4
	2	175	47.9	2.9	40.5–51.7	26.0	1.5	22.0–28.7
	3	14	51.4	1.8	48.8–54.7	27.9	2.0	25.0–31.7

*Notes*: The dimensions were calculated across samples per cesspit. For *Ascaris* sp., fertilised and unfertilised eggs were distinguished. For *Ascaris* sp. and *Trichuris* sp., measurements were from the first 100 eggs viewed. For *Capillaria* sp., *Dicrocoelium* sp., *Fasciola* sp. and *Taenia* sp., all measurable eggs were included to calculate dimensions.

Cesspit 1 yielded three eggs of the genus *Taenia* in two different samples. Morphologically, the Asian tapeworm (*T. asiatica*), the beef tapeworm (*T. saginata*) and the pork tapeworm (*T. solium*) are not distinguishable (Garcia, 2016). However, as *T. asiatica* is endemic to Asia, it is more likely these eggs with their characteristic radially striated shell and six-hooked oncosphere represent either beef or pork tapeworm. The dimensions of all three eggs were well within the modern size range of 30–47 *μ*m (Garcia, 2016; Mehlhorn, 2016; Pümpin *et al*., 2017). The mean length of eggs in Cesspit 1 was 36.2 *μ*m and mean width was 34.1 *μ*m.

Two *Capillaria* sp. eggs were found in Cesspit 2, within layer US20-134. These eggs are barrel-shaped with polar plugs, and striations or pores on the outer shell. The shape resembles that of *T. trichiura* but the striated surface is what allowed for distinction. Furthermore, they differ in size at 40–67 *μ*m in length and 27–35 *μ*m in width. The species of *Capillaria* could not be determined from microscopy alone. However, the pores were irregularly dispersed and rounded, which would fit with the ‘punctuated’ morphotype described by other authors (Maicher *et al*., 2017).

### ELISA

Using ELISA analysis, *G. duodenalis* was present in all three cesspits, *E. histolytica* was found in Cesspits 1 and 2, while *Cryptosporidium* was absent in all cesspits (Table 1). On the first ELISA test, multiple wells tested positive for *G. duodenalis* for all samples from Cesspit 1 except for sample US4139(457) which had no positive wells. Similarly, multiple wells tested positive for *G. duodenalis* from both samples from Cesspit 2 and from the sample from Cesspit 3. Similar results were obtained on the retest using a different subsample of sediment from the cesspits on a later date.

For *E. histolytica*, two of the four samples from Cesspit 1 had positive wells on the first and second ELISA tests. One of the two samples from Cesspit 2 had positive wells on both tests and no wells were positive for the sample from Cesspit 3.

## Discussion

This study has identified eight species of intestinal parasite as being present in Brussels during the late Medieval and Renaissance periods. Some would have been pathogenic to a significant proportion of the inhabitants (in particular dysentery), others may have caused problems in susceptible groups such as children (especially roundworm and whipworm), while the remainder may indicate false parasitism from the consumption of animal organs. We will now consider how these new data improve our understanding of health and disease in the population, whether there was a notable variation in parasitism between different family groups, and if we can identify change between the Medieval period and the Renaissance.

### Which parasites dominated?

Whipworm and roundworm, together with the protozoa *E. histolytica* and *G. duodenalis*, were found throughout the population. Our identification of Medieval *G. duodenalis* is of note, as the previous earliest published evidence for Belgium is at 18th-century Namur (Gonçalves *et al*., 2002). Whipworm, roundworm and protozoa that cause dysentery have been infecting humans throughout our evolution, but became more common as population densities increased with urbanization (Mitchell, 2013, 2017). The egg concentrations for *Trichuris* sp. and *Ascaris* sp. were many orders of magnitude higher than was the case for the zoonotic parasites such as *Taenia* sp. and *Fasciola* sp. Egg counts of 40 000 eggs g^−1^ are rare in archaeological contexts, and indicate both excellent preservation conditions and also a high intensity of infection in the original population. To put this in a clinical context, an egg concentration of >9999 eggs g^−1^ in a fresh fecal sample is categorized by the WHO as representing a high whipworm burden (World Health Organization, 1991). In contrast, the highest concentration of roundworm eggs recorded was 14 555 (US33-15, Cesspit 3), which falls at the lower end of the medium infection burden range: 5000–49 999 eggs g^−1^ (World Health Organization, 1991). Palaeoparasitological latrine soil analyses for Medieval samples from northern Europe have frequently found higher concentrations of *Trichuris* sp. eggs than of *Ascaris* sp. (Yeh *et al*., 2014; Rácz *et al*., 2015). It is important to remember that cesspit samples contain feces from multiple individuals deposited over time, and many of the original eggs may have been destroyed by fungi and insects, so archaeological egg counts are not directly equivalent to egg count data from modern fresh fecal samples. Nevertheless, these findings would suggest that parasites spread by the fecal contamination of food and drink were the dominant group in this population.

When we compare the species of parasite present in Medieval and Renaissance Belgium with other regions, we can see that there are similarities with other towns in northern Europe, but clear contrasts with southern Europe during the same period (Mitchell, 2015). In southern Europe, whipworm and roundworm were fairly common in the Medieval period, but zoonotic parasites were rare (Knorr *et al*., 2019). It is likely that a combination of the contrasts in both climate and the dietary habits of northern and southern Europeans was responsible for this contrast. The hot weather in the south meant Mediterranean populations found fresh meat and fish went rotten quicker than was the case in northern Europe, so dishes of raw and pickled meat and fish were less common in the south than the north.

#### Differentiating true infection from false parasitism

Liver flukes such as *D. dendriticum* and *F. hepatica* tend to reside in the bile ducts of herbivores, including those regularly consumed by humans such as cattle, sheep, goat and deer (Garcia, 2016). Human infection with *Fasciola* commonly occurs when aquatic vegetation with encysted metacercariae is consumed without sufficient cleaning or cooking (Mas-Coma *et al*., 2018). True infections of *D. dendriticum* occur when its secondary intermediate host, the ant, is ingested accidentally, as they can be found on a variety of edible plants and herbs. Eggs from these two parasites can also be found in human feces in cases of spurious infection (false parasitism), after eating the uncooked liver of animals infected by the parasite (Garcia, 2016). Low concentrations of eggs from both species were identified in our samples, suggesting that active infections amongst multiple individuals using the cesspits were unlikely, and either a few individuals were lightly infected or false parasitism was present from eating the undercooked organs of infected animals.

The main hosts of *Capillaria* sp. are rodents and other carnivores. Infected animals have eggs in their feces that embryonate in the environment after approximately 5–8 weeks under damp conditions (Fuehrer, 2011). In the most common species known to infect humans (*C. hepatica*), the presence of eggs in human feces represents false parasitism, since in a true infection the worms deposit eggs in the liver that are not released in the feces (Govil and Desai, 1996). This suggests that either humans using the cesspit were eating infected animal liver, or that the eggs may be present as a consequence of rodent feces ending up in the cesspit.

#### Variation between households

Our results show that whipworm and roundworm were consistently found in the three cesspits. Egg counts were also high in each cesspit. Some layers had higher or lower levels than others (see e.g. US24-122), and this may well result from the sampling levels including non-fecal layers rather than indicating a period of use by people not infected by parasites. For protozoa, again all three cesspits were positive for *G. duodenalis*, and two positive for *E. histolytica*. The negative *E. histolytica* result for Cesspit 3 may genuinely indicate its absence from those using that latrine, or result from the fact that only one sample was available for us to analyse, in contrast to the multiple layers tested in the other cesspits.

Zoonotic parasites were present with much lower egg counts. None were found in every sample, or even in every latrine. The low egg counts across the three cesspits would again indicate similarities in all users, in that zoonotic parasites were present but rare, and false parasitism from *Capillaria* sp. and *Dicrocoelium* sp. eggs was present but rare.

The patterns seen would fail to suggest evidence for notable variation in the parasite infection in different households within the city. Similarly, there was no clear evidence for change over time, with a similar pattern in the 14th–15th century and the 15th–17th century latrines. It may well be that fecal–oral parasites were common in the whole population, and zoonotic parasites present but uncommon in the whole population.

### Way of life in Brussels revealed by intestinal pathogens

The health consequences of parasites in Medieval and Renaissance Brussels would vary considerably with the parasite involved. *Entamoeba histolytica* and *G. duodenalis* will cause dysentery in a proportion of those infected, and some of those would have died, especially children (Marie and Petri, 2013). Roundworm and whipworm contribute to chronic morbidity, leading to malnutrition, stunting of growth and impaired cognitive development in some children (Dold and Holland, 2011; Else *et al*., 2020). Children would have likely been particularly badly affected as they tend to be less able to tolerate intestinal parasites, and can develop vitamin deficiencies and impaired growth (World Health Organisation, 2002). At times when food supply was low, these problems would have been more severe, possibly leading to nutrient deficiencies in those with the greatest parasite loads. Polyparasitic infections would increase this risk (Sanchez *et al*., 2013; Al-Delaimy *et al*., 2014).

The presence of fecal-orally spread helminths and dysentery cysts increases the likelihood that other infectious disease agents (which would not have been preserved) with the same manner of spread could have been present. Enteroviruses, hepatitis A, rotavirus, *Campylobacter jejuni*, *Escherichia coli*, *Salmonella* and *Shigella* have all been identified as non-parasite pathogens that can be spread by fertilizing with human fecal material (Blum and Feachem, 1985: 5). It is likely that inhabitants of Brussels in the late Medieval and Renaissance periods may have also suffered from the effects of some of these infections.

Brussels was long notorious for its slow-flowing, putrid city river, the Senne (Deligne, 2015). The high concentration of fecal-orally transmitted parasites found in all three cesspits is likely linked to this. Prior to the beginnings of the city's sewage system in the 17th century, waste (including that from cesspits) was generally dumped into the Senne directly or onto the streets, where it would eventually discharge into the river. Sewage was routinely deposited into the Senne well into the 18th century (Deligne, 2003, 2016). Plans for broad irrigation with wastewater in peri-urban agricultural areas existed in the early 19th century, but were abandoned by the second half of the 19th century (Kohlbrenner, 2014). The Senne again came to represent the sewer of the Belgian capital, with wastewater purification only introduced in the 20th century. The heavily contaminated river, which routinely flooded surrounding neighbourhoods including that of Cesspit 1 as indicated by excavation reports, also represented a source of water that could be used by poor households. Indeed, the wells, water points and fountains on public streets implemented by the city as sources of water supply by the late 13th century (Petit, 2016) represented a more symbolic than functional system until the 17th century (Deligne, 2015).

The use of human feces as fertilizer for agriculture in the vicinity of Brussels after the 13th century was also likely to have been an important source of fecal contamination of food consumed in the city (Devos *et al*., 2009, 2013; Vannieuwenhuyze *et al*., 2015). This form of manuring was a common practice in Medieval Europe (Magnusson, 2013). Abattoir waste was also used, creating a possible avenue for transmission of zoonotic parasite species (Ledger and Mitchell, 2019). It is likely that the fecal waste would be directly applied to fields rather than first composted for many months. This period of composting is necessary to significantly decrease transmission of whipworm and roundworm (Ziegelbauer *et al*., 2012; Strunz *et al*., 2014). The use of human fecal material that is not correctly composted still poses a major health threat today in rural areas of developing countries due to soil-transmitted helminths including roundworm and whipworm (Blum and Feachem, 1985; Phuc *et al*., 2006).

*Taenia* sp. eggs were recovered in 14th–15th century Brussels, and were present in 11th–13th as well as 15th–17th century Namur (Rocha *et al*., 2006). We were not able to distinguish between the two species of *Taenia* tapeworm based on the morphology of identified eggs, as ancient DNA analysis of these materials would be required to identify which species of tapeworm was present at the site (Anastasiou and Mitchell, 2013*b*; Søe *et al*., 2018). The finding suggests at least partial dietary consistency in the broader region, with raw or undercooked beef and/or pork having been consumed in the city. While Cesspit 1 was close to a cattle abattoir (Billen, 1997; Charruadas *et al*., 2017), we know that both pork and sheep were more commonly eaten meats in Late Medieval Europe (Doehaerd, 1982; Polet and Katzenberg, 2002; Ervynck and Neer, 2017).

Given the importance of the river Senne not just for Brussels itself, but also for peri-urban agricultural and piscicultural areas (Deligne, 2015), the absence of fish tapeworm (*Dibothriocephalus* sp.) in the analysed subsamples from all three latrines is notable. *Dibothriocephalus* sp. is a parasite frequently found in palaeoparasitological contexts where freshwater fish, an intermediate host for the parasite, figures as an important dietary component (Garcia, 2016). Given records for intense pisciculture of carp in 14th century Brussels (Deligne, 2003), and abundant archaeological evidence for fish remains, both marine, anadromous and freshwater, from late Medieval Brussels, including the Kartuizerstraat cesspits (Van Neer, 2001; Charruadas *et al*., 2017; De Cupere *et al*., 2018), thorough cooking of fresh fish seems the most likely interpretation.

## Conclusion

This study analyses multiple latrines from the same city in order to assess diversity in parasitism between households, and to place this knowledge in the context of the lives of those who lived in Brussels during the late Medieval and Renaissance periods. High concentrations and diversity of fecal-orally transmitted parasites highlight the sanitation conditions in the city, enabling the spread of roundworm, whipworm and pathogenic protozoa that cause diarrhoea and dysentery (*E. histolytica* and *G. duodenalis*). Drinking water from the river Senne, river flooding and manuring of market gardens with human feces are all likely to have contributed to the spread of these parasite species. Eating raw or undercooked liver may have resulted in the presence of *Capillaria* sp., *Fasciola* liver fluke and Lancet liver fluke. While any consumed fish was likely thoroughly cooked as reflected in the absence of the fish tapeworm, pork and/or beef was not always adequately cooked, resulting in pork and/or beef tapeworm infections. Our study also represents the earliest paleoparasitological finding of Lancet liver fluke as well as of *G. duodenalis* in the area corresponding to modern-day Belgium. We found little evidence for notable diversity in parasite species between households, or between the late Medieval and Renaissance periods. This suggests a stable pattern of infection among the population of the city, dominated by poor sanitation.
